# Structural MRI at 7T reveals amygdala nuclei and hippocampal subfield volumetric association with Major Depressive Disorder symptom severity

**DOI:** 10.1038/s41598-019-46687-7

**Published:** 2019-07-15

**Authors:** S. S. G. Brown, J. W. Rutland, G. Verma, R. E. Feldman, J. Alper, M. Schneider, B. N. Delman, J. M. Murrough, P. Balchandani

**Affiliations:** 1 Translational and Molecular Imaging Institute, Icahn School of Medicine at Mount Sinai, New York, New York United States; 2 Mood and Anxiety Disorders Program, Department of Psychiatry, Icahn School of Medicine at Mount Sinai, New York, New York United States; 3 Department of Radiology, Icahn School of Medicine at Mount Sinai, New York, New York United States; 4 Department of Neuroscience, Icahn School of Medicine at Mount Sinai, New York, New York United States

**Keywords:** Cognitive neuroscience, Predictive markers, Amygdala

## Abstract

Subcortical volumetric changes in major depressive disorder (MDD) have been purported to underlie depressive symptomology, however, the evidence to date remains inconsistent. Here, we investigated limbic volumes in MDD, utilizing high-resolution structural images to allow segmentation of the hippocampus and amygdala into their constituent substructures. Twenty-four MDD patients and twenty matched controls underwent structural MRI at 7T field strength. All participants completed the Montgomery-Asberg Depression Rating Scale (MADRS) to quantify depressive symptomology. For the MDD group, volumes of the amygdala right lateral nucleus (*p* = 0.05, *r*^*2*^ = 0.24), left cortical nucleus (*p* = 0.032, *r*^*2*^ = 0.35), left accessory basal nucleus (*p* = 0.04, *r*^*2*^ = 0.28) and bilateral corticoamygdaloid transition area (right hemisphere *p* = 0.032, *r*^*2*^ = 0.38, left hemisphere *p* = 0.032, *r*^*2*^ = 0.35) each displayed significant negative associations with MDD severity. The bilateral centrocortical (right hemisphere *p* = 0.032, *r*^*2*^ = 0.31, left hemisphere *p* = 0.032, *r*^*2*^ = 0.32) and right basolateral complexes (*p* = 0.05, *r*^*2*^ = 0.24) also displayed significant negative relationships with depressive symptoms. Using high-field strength MRI, we report the novel finding that MDD severity is consistently negatively associated with amygdala nuclei, linking volumetric reductions with worsening depressive symptoms.

## Introduction

Major depressive disorder (MDD) is a chronic and debilitating psychiatric illness characterized by numerous of symptoms, including, low mood, anhedonia, changes in appetite, hyper- or hyposomnia, concentration difficulties and suicidality^1^. At present, MDD is one of the leading causes of disability worldwide, with reports suggesting that one in six adults will suffer from MDD at some point during their lifetime^2^. MDD has a complex etiology, with a complex multifactorial interaction of genetic, neurobiological, and environmental components likely contributing to the individual differences seen in clinical liability and depressive symptomology^1^. To illustrate, molecular genetics investigations report narrow-sense heritability estimates of 37%^3,4^, suggesting a significant genetic component. Epidemiological research has found strong association between MDD and factors such as socioeconomic status, environmental stressors and neuroticism^5,6^. Further evidence from neurobiological studies suggests that the temporal limbic system is critical to the onset of MDD, due to its importance in emotion regulation, stress response, enhanced plasticity, and its sensitivity to MDD polygenic risk^7–11^.

Reported volumetric data focused on the hippocampal and amygdala structures in MDD have been notably inconsistent. Larger^12,13^, smaller^14^, and no significant differences^15^ in limbic volumes have all been reported in MDD cohorts compared to control subjects. Where amygdala findings appear to be more diverse, meta-analyses of large datasets point to reduced hippocampal grey matter volume being a commonly replicated finding in MDD^16,17^. Moreover, smaller hippocampi in major depression have frequently been demonstrated to be moderated by age of onset^17–19^. Heterogeneity within and between sample groups and significant confounding factors have been cited as reasons for disparate findings. One such confounding factor is the varying proportions of depressed patients currently taking antidepressant medication at the time of study^20^. Findings of hippocampal and amygdala volumetric deficits at the hippocampus and amygdala in MDD have often been attributed to excitotoxicity from elevated subcortical metabolic activity, which is consistent with increased activation at the amygdala and anterior cingulate cortex during negative stimuli processing on functional MRI^21,22^. In animal studies, depressive and stressed phenotypes are associated with reduced hippocampal neurogenesis, brain-derived neurotrophic factor (BDNF) levels and dendritic branching^23–25^ and antidepressant medication has been shown to inhibit these stress-induced processes while promoting neurogenesis^26^. Immunohistochemistry carried out on human hippocampi revealed a significantly increased number of neural progenitor cells and capillary area in dentate gyri of MDD patients on serotonin re-uptake inhibitor medication compared to both control subjects and unmedicated MDD patients, and furthermore that dentate gyrus volume was directly correlated with neural progenitor cell numbers^27^. Longitudinal assessment in humans has also shown that citalopram treatment over an 8 week period is associated with a regional increase in hippocampal grey matter^28^. Moreover, meta-analyses identified that significant enlargements of the amygdala are specifically associated with positive medication status in MDD patients compared to healthy controls, whereas unmedicated MDD patients exhibited decreased amygdala volumes^29^.

The amygdala and hippocampal formation are commonly treated as single entities in structural MRI in humans, however *ex vivo* high resolution human imaging and animal histological studies show that they are comprised of distinct substructures^30–32^. The amygdala is known to be comprised of multiple nuclei which exhibit differing connectivity and cellular profiles^30,32–35^, and the hippocampus is comprised of the cornu ammonis (CA), dentate gyrus and subiculum^30^. These regions have been shown in disease models to react differentially to pathological mechanisms, as well as having diverse functions in non-disease states^21,36–38^. It is therefore a prescient question in human *in vivo* MRI investigation which particular amygdala nuclei and hippocampal subfields are affected in major depression, and whether substructures are differentially affected. Much of the existing research on structural MRI in depression has utilized manual tracings of the amygdala and hippocampus^8,20,21,39,40^. Whilst manual tracing is regarded as the gold standard for anatomical precision and accuracy, individuals may vary in their tracing conventions, meaning comparisons between different investigator groups could be impaired in terms of replicability. Additionally, variability in spatial resolution and slice thickness have been implicated in differences between measurements, as these parameters may affect boundary demarcation of anatomical regions^41^. With the emergence of automated segmentation techniques, the feasibility and time commitment of investigating subcortical substructures in larger datasets is improved.

To the author’s knowledge, 7 Tesla MRI has not been leveraged to segment the amygdala in any clinical population. Hippocampal segmentation has become a recent prominent focus of ultra high-field MRI, the techniques and possible applications of which have been reviewed by Giuliano *et al*.^42^. The present study aimed to expand upon existing findings of whole subcortical volumes in major depression by utilizing the enhanced resolution of ultra high-field strength MRI and a computational segmentation atlas^43^. Recent findings are suggestive that hippocampal CA1 volume in particular is a potential biomarker of MDD status^44^. Here, we aimed to examine hippocampal subfield volumes at enhanced field strength, in addition to investigating whether, in a similar manner to subfield susceptibility, the amygdala nuclei displayed subregion-specific changes related to MDD. To minimize the aforementioned methodological issues of previous reports, we limited our population to an MDD patient cohort currently not taking antidepressant medication to investigate the association between (1) hippocampal subfield and amygdala nuclei volume (2) duration of illness and 3) MDD symptom severity. FreeSurfer (http://surfer.nmr.mgh.harvard.edu) automated segmentation of the volumes was used to eliminate intra- and inter-rater bias of manual tracing and maximize reproducibility^43^.

## Methods

### Subjects

Twenty-four participants (mean age = 39.6, SD = 10.4, 9 females) with a primary diagnosis of MDD were recruited through the Mood and Anxiety Disorders Program at the Icahn School of Medicine at Mount Sinai to take part in a pilot analysis. Twenty age-matched controls were also recruited (mean age = 39.5, SD = 12.5, 5 females). All participants were English-speaking and between 18 and 65 years of age. Age was not significantly different between groups (*p* = 1.0). Eligible patients had a primary diagnosis of major depressive disorder, without psychotic features, assessed by the Structured Clinical Interview for DSM-IV disorders (SCID-IV) or the Structured Clinical Interview for DSM-5 Research Version (SCID-5-RV)^45,46^. They were antidepressant free for at least 4 weeks prior to study participation and were currently experiencing a major depressive episode. No depressed participants had previously undergone electroconvulsive therapy and none were known to have a co-morbid anxiety disorder. Eleven of the MDD participants had undergone unsuccessful antidepressant treatment in their lifetime, 6 of these individuals receiving more than one unsuccessful drug. History of concussion or head injury was unknown for the sample. Healthy controls had no current or lifetime psychiatric disorder as determined by the SCID-IV or SCID-5-RV^45,46^. Participants with a current diagnosis of obsessive compulsive disorder (OCD), alcohol or substance abuse in the previous year, or lifetime history of a psychotic illness, bipolar disorder, or neurological disease were excluded. Participants with MRI contraindications, unstable medical conditions, or positive urine toxicology on day of scan were also excluded.

Depressive symptomology and severity was assessed using the Montgomery-Asberg Depression Rating Scale (MADRS; range 0–60; higher score indicates greater depression severity). The SCID and MADRS were all administered by trained clinical raters at screening, within 4 weeks of the MRI scan. The depressed sample had a mean duration of illness (current episode) of 80.7 months (SD = 85.3 months) and a mean age of onset of 17.6 years (SD = 10.4). All participants gave fully-informed written consent prior to investigation. This protocol was approved by the local Institutional Review Board, the Human Research Protection Program at the Icahn School of Medicine at Mount Sinai. All methods were performed in accordance with the relevant guidelines and regulations.

### MRI acquisition

Structural MRI data was acquired for all participants on a 7 Tesla whole body scanner (Magnetom, Siemens Healthcare, Erlangen, Germany). A SC72CD gradient coil was used with a single coil transmit and a 32-channel head coil (Nova Medical, Wilmington, MA, USA). A T1-weighted MP2RAGE sequence was performed on each participant, with a 0.7 mm × 0.7 mm × 0.7 mm voxel resolution. Field of view (FOV) was 225 × 183, orientation of scan was coronal, repetition time (TR) was 6000 ms and echo time (TE) was 3.62 ms. A coronal-oblique T2-weighted turbo spin echo (T2-TSE) sequence was also obtained for all participants, with a 0.43 mm × 0.43 mm × 2.0 mm voxel resolution. FOV was 222 × 177, orientation of scan was coronal, TR was 9000 ms and TE was 69 ms.

### Amygdala and hippocampal segmentations

**Figure 1:**
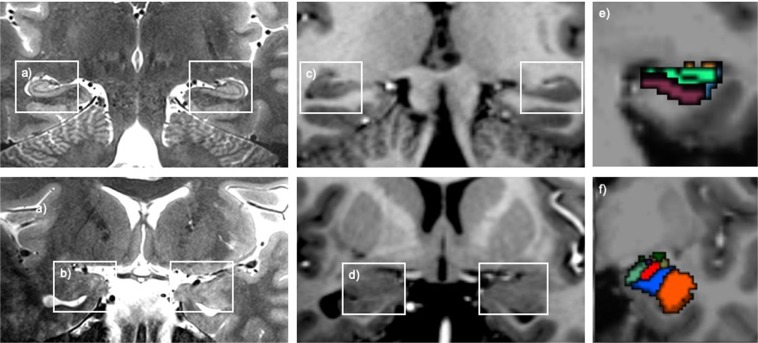
(**a**) T2-weighted images in the coronal orientation showing visibility of the hippocampus and (**b**) the amygdala at a resolution of 0.21 × 0.21 mm. (**c**) T1-weighted images in the coronal orientation showing visibility of the hippocampus and (**d**) the amygdala at an isotropic resolution of 0.7 mm. (**e**) Example coronal slice of the hippocampal subfield segmentation and (**f**) the amygdala nuclei segmentation.

Image reconstruction and automated segmentation of the whole amygdala into subnuclei and the whole hippocampus into subfields was carried out in FreeSurfer (http://surfer.nmr.mgh.harvard.edu) version 6.0. The amygdala segmentation algorithm is based on Bayesian inference, and was developed using ten *ex vivo* human hemispheres, scanned at 7T field strength with a isotropic spatial resolution of 0.1 mm. Verification of substructures was carried out by a neuroanatomist and the segmentation was validated for performance using the publicly available ADNI and ABIDE neuroimaging datasets^43^. Similarly, the hippocampal subfield segmentation algorithm is built using Bayesian inference methodology, using data from fifteen 0.13 mm isotropic resolution autopsy scans and was validated using the ADNI dataset^47^. The segmentation processes use statistical inference to identify subregions of interest. Visual representation of the available level of anatomical detail in the present study is shown in Fig. 1.

**Figure 2:**
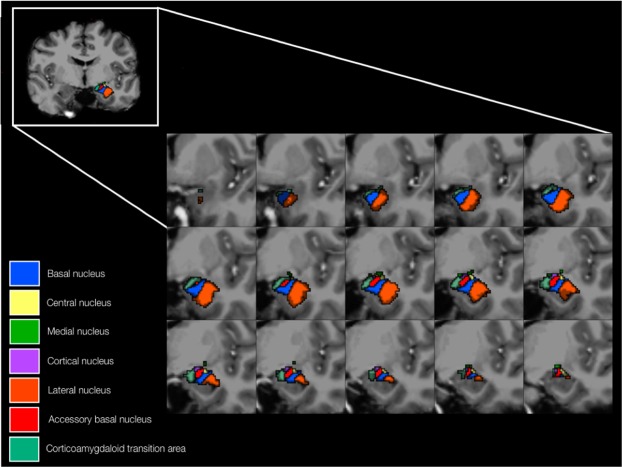
An anatomical representation of the segmented amygdala nuclei, including the superficial structures (central, medial and cortical nuclei), the deep structures (basal, lateral and accessory basal nuclei) and the corticoamygdaloid transition area. The volumes of the lateral nucleus, cortical nucleus, accessory basal nucleus and corticoamygdaloid transition area displayed significant negative associations with MDD severity.

**Figure 3:**
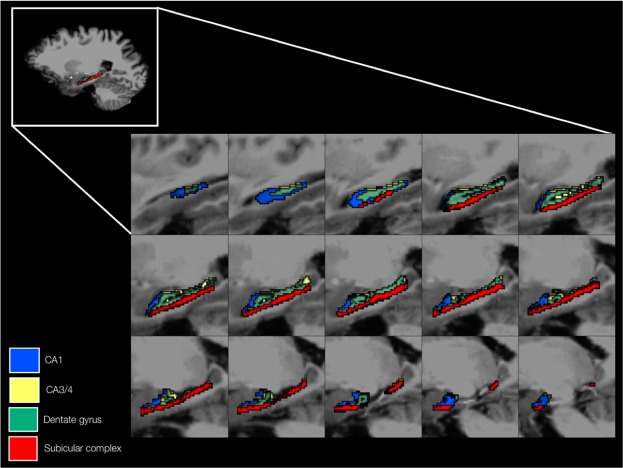
An anatomical representation of the hippocampal subfields, with the subiculum, presubiculum, parasubiculum, CA1, CA3, CA4, granule layer of the dentate gyrus and molecular layer of the dentate gyrus grouped into CA1, CA3/4, dentate gyrus and subicular complex regions. Volume metrics of CA1 and CA3/4 exhibited negative associations with MDD severity.

Both T1- and T2-weighted images were utilized to maximize accuracy of the segmentation process. All FreeSurfer outputs were manually inspected for quality, segmentation accuracy and correct co-registration during the analysis. The amygdala was segmented into the lateral, basal, accessory basal, cortical, medial and central nuclei and the corticoamygdaloid transition area (Fig. 2). The superficial structures and the deep structures were also investigated as the centrocortical complex (central, medial and cortical nuclei) and the basolateral complex (basal, lateral and accessory basal nuclei) respectively. The hippocampus was segmented into the subiculum, presubiculum, parasubiculum, CA1, CA3, CA4, the granule cell layer of the dentate gyrus, the molecular layer of the dentate gyrus, the hippocampal-amygdala transition area and the fimbria. Subfields were combined into CA1, CA3/4, the subicular complex (pre-, para- and subiculum) and the dentate gyrus (granule cell layer and molecular layer) to ensure subfield structures were large enough for accurate volume quantification (Fig. 3).

### Statistical analysis

Amygdala nuclei and hippocampal subfield volumes were normalized to intracranial volume (ICV). Normalization was favored over covariation in this case to be prudent, as ICV trended towards being lower in the MDD group (*p* = 0.2). Between group volumetric analyses were carried out using two-tailed independent t-testing, adjusting for age and gender. Linear regression analyses, also controlled for age and gender by the addition of the variables into the linear model as covariates, were performed on the MDD group only. Multiple comparisons were corrected for using false-discovery rate (FDR) on the *p* value outputs, accounting for separate testing of hippocampal subfields and amygdala nuclei. Orthogonalization was carried out on the data with a principle component analysis implemented using the generic ‘princomp’ function in R, computing eigenvalues and eigenvectors from the correlation matrix derived from the structural data and MADRS scores. All statistical analyses were carried out in R version 3.3.3. (https://www.r-project.org/). Significance level was assumed at p < 0.05 and determination coefficients are adjusted *r*^*2*^.

## Results

### Amygdala nuclei

Volumetric changes in the amygdala nuclei were t-tested, adjusting for age and gender. We did not find evidence to support the hypothesis that nuclei volume normalized to ICV are different between groups (Table 1).

**Table 1:** Between group statistical analysis results for amygdala nuclei, whole amygdala, hippocampal subfields and whole hippocampus volumes.

Subcortical region	Left hemisphere		Right hemisphere	
	*p*	Cohen’s *d*	*p*	Cohen’s *d*
Corticoamygdaloid transition area	0.97	0.29	0.92	0.41
Lateral nucleus	0.95	0.27	0.97	0.35
Basal nucleus	0.90	0.44	0.92	0.41
Accessory basal nucleus	0.95	0.39	0.93	0.49
Central nucleus	0.93	0.54	0.90	0.49
Cortical nucleus	0.95	0.32	0.93	0.34
Medial nucleus	0.95	0.05	0.92	0.29
Basolateral complex	0.84	0.36	0.79	0.40
Centrocortical complex	0.81	0.32	0.82	0.46
Whole amygdala	0.84	0.35	0.81	0.17
CA1	0.92	0.05	0.92	0.52
CA3/4	0.92	0.10	0.90	0.66
Subicular complex	0.92	0.20	0.88	0.09
Dentate gyrus	0.92	0.72	0.90	0.39
Whole hippocampus	0.64	0.09	0.96	0.07

**Figure 4:**
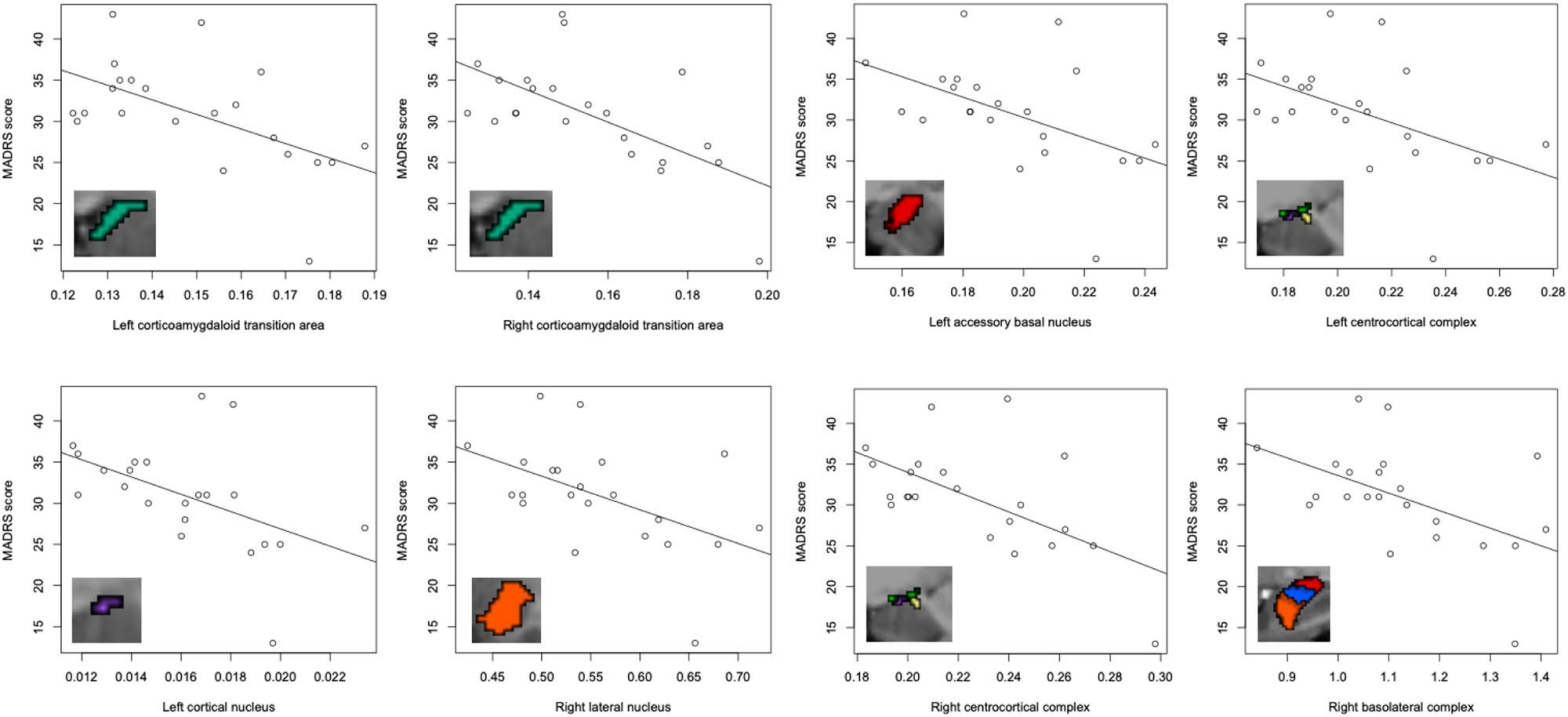
Regression plots of the identified significant negative associations, surviving FDR correction, between amygdala nuclei and depressive symptoms rated by the MADRS.

Regression analyses in the MDD group, adjusted for age and gender, were used to investigate possible associations between amygdala nuclei volumetrics with MDD episode duration and severity, as quantified by MADRS score (Fig. 4). Significant negative associations of volumetric measurement with MADRS-rated depression symptomatology at both corticoamygdaloid transition areas (left hemisphere *p* = 0.032, *r*^*2*^ = 0.35, right hemisphere *p* = 0.032, *r*^*2*^ = 0.38), right lateral (hemisphere *p* = 0.050, *r*^*2*^ = 0.24), left accessory basal (*p* = 0.040, *r*^*2*^ = 0.28), left cortical (*p* = 0.032, *r*^*2*^ = 0.35) were found. When grouped, both centrocortical complexes (left hemisphere *p* = 0.032, *r*^*2*^ = 0.32, right hemisphere *p* = 0.032, *r*^*2*^ = 0.31) and the right basolateral complex (*p* = 0.050, *r*^*2*^ = 0.24) also showed a significant negative relationship with MADRS score. All reported *p* values are FDR corrected. The right accessory basal nucleus and right basal nucleus also showed associations with MDD severity, however the findings did not remain significant when the analysis was adjusted for age (*p* = 0.059, *r*^*2*^ = 0.18) and gender (*p* = 0.073, *r*^*2*^ = 0.16). Mean volumetrics upon grouping into mild, moderate and severe MADRS scores are presented in Supplementary Fig. S1. No significant associations were observed between amygdala nuclei volume and MDD duration. Additional analyses were carried out with ICV as a covariate in the regression model, in place of using normalization based on region of interest to ICV ratio. There were no changes to statistical significance between these two methods of controlling for ICV.

### Hippocampal subfields

Potential volumetric differences of the hippocampal subfields across study groups were investigated, adjusting for age and gender. There were no significant between group differences identified (Table 1).

**Figure 5:**
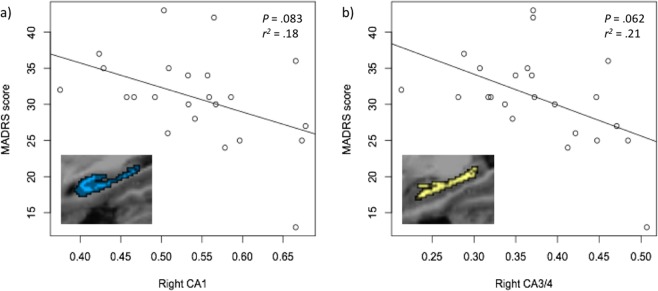
Regression plots of the associations between hippocampal subfields CA1 and combined CA3/4 and depressive symptoms rated by the MADRS.

Regression analyses (adjusted for age and gender) revealed associations between MDD severity and volume of the right CA1 subfield and the right CA3/4 subfields, however these did not survive correction for false discovery (*p* = 0.083, *r*^*2*^ = 0.18 and *p* = 0.062, *r*^*2*^ = 0.21 respectively) (Fig. 5). Mean volumetrics upon grouping into mild, moderate and severe MADRS scores are presented in Supplementary Fig. S1. There were no significant associations between subfield volumetrics and duration of illness. Analyses adding ICV as a covariate into the regression model instead of using a ratio-based normalization did not influence statistical significance.

### Principle component analysis

Interdependence of the volumetric nuclei and subfield measurements was assessed using principle component methodology. Variables above average contribution for the first component, where average contribution is defined as 1/length(variables), were left whole amygdala, left lateral nucleus, left basal nucleus, right lateral nucleus, left accessory basal nucleus, left corticoamygdaloid transition area, left subicular complex, right accessory basal nucleus, right CA1, right subicular complex, left CA1, left central nucleus and left dentate gyrus. For component 1, proportion of variance accounted for was 58.1%. Significantly contributing variables to the second component were MADRS score, group, right central nucleus, right CA3/4 and left medial nucleus, accounting for 9.1% of data variance. Component 3 comprised of the right medial nucleus, right central nucleus, right cortical nucleus, MADRS score, group, right accessory basal nucleus and left dentate gyrus, with a proportion of explained variance of 7.3%.

## Discussion

To our knowledge, this is the first high-field MRI investigation that leverages enhanced signal from 7T scanning to examine amygdala nuclei and hippocampal subfield volumetrics in Major Depressive Disorder. MDD participants showed significant sensitivity of subcortical subregion volumes to depression severity. However, no significant volumetric changes in MDD compared to controls were identified when investigating hippocampal subfields, amygdala nuclei or whole structures with stringent control for the known effects of age, gender and antidepressant treatment.

Our present high-field imaging analysis of the amygdala shows that in the left hemisphere, the centrocortical complex (driven mainly by the cortical nucleus), the accessory basal nucleus and the corticoamygdaloid transition area are significantly associated with MDD severity. In the right hemisphere, all regions were significantly related to MDD symptomatology, although some nuclei only exhibited correlations when grouped into the basolateral and centrocortical complexes. Moreover, orthogonalization of the data showed that although many subregions of the amygdalo-hippocampal complex are cross-correlated, component loadings did not tally with volumes of significant association with MADRS score. This indicates that the present results do not reflect a general sensitivity of the overall complex to depression severity, but show changes of individual nuclei that are to some degree independent.

The amygdala sub-complexes and nuclei are considered anatomically, functionally, and connectively separate^35,48,49^, and previous studies of mood disorders and stress in animal models show differential GABAergic system modulatory responses of the amygdala nuclei to specific mood disorder-implicated behaviors, linking the basolateral complex to fear extinction, the medial nuclei to sociability and the central nuclei to anxiety and aggression^50–54^. Nevertheless, in rat models of stress, markers of neuronal inhibition in the amygdala were reduced uniformly across nuclei compared to controls. Evidence from animal models also shows that multiple amygdala nuclei exhibit associations between grey matter volume and depressive phenotypes^55^. The results reported here concur with a volumetric sensitivity of many amygdala substructures to depression symptom severity in humans, as opposed to vulnerability of a few specific nuclei. However, the literature of amygdala volume differences and association to depressive symptoms remains inconsistent. It is possible that during the development of the depressive phenotype amygdala volume may follow a trajectory of change which is shown at different relative time points by variation between study populations. This is somewhat supported by our data, as classifying MADRS symptom severities into mild, moderate and severe in our sample revealed that mild and moderate severities frequently exhibit a qualitative increase in regional volumes, which may be a causative factor in why a significant negative association was found between MADRS scores and amygdala nuclei volumes, but no significant between group difference was identified. Additionally, it is plausible that as the increased spatial resolution of this 7T study allows a greater degree of granular separation of amygdala nuclei, it therefore reflects a more detailed picture than amygdala volume as a whole. A previous study of *in vivo* parcellation of the human amygdala in healthy individuals showed that although the intra-amygdala regions appear mostly uniform at 3T field strength, notably increased signal-to-noise ratio at 7T allows for consistent segmentations of the amygdala based on structural image intensities^52^. Similarly, a 3T and 7T comparison study concluded that the amygdalo-hippocampal border, formed by cerebrospinal fluid in the temporal horns of the lateral ventricles and the thin sheet of white matter known as the alveus, was visualized with considerably higher detail at increased field strength^53^.

Regression analyses also suggested a relationship between hippocampal subfields CA1 and CA3/4 and depressive symptom severity, however these results did not survive correction for multiple comparisons. Nevertheless, in a relatively small sample size, this uncorrected association remains promising. Previous studies have found similar relationships at the whole hippocampal structure level, with number of depressive episodes negatively correlating with grey matter volume in the right hippocampus and amygdala using voxel-based morphometry (VBM) methodology^56^.

Our results suggest that MDD severity has a significant effect on multiple individual amygdala nuclei volumes, in addition to the superficial and deep grouped nuclei structures, and to a lesser degree, hippocampal subfields. A popular theory of the impact of stress and depression on reducing limbic volumes is neural excitotoxicity mediated by hypothalamic pituitary adrenal (HPA) axis hyperactivity and associated reduced levels of BDNF^24,25,57,58^. BDNF promotes survival, maturation and differentiation of neurons and synaptic connectivity, especially in the basal forebrain and cortex^59^, its signaling has been implicated in antidepressant efficacy^60,61^ and BDNF serum reductions have been associated with intense stress states, social defeat, despair behavior and reduced hippocampal neurogenesis and volume^62,63^. Along with excess corticosterone, it is possible that a combination of diminished neuronal survival promotion and stress-mediated overactivity may lead to a loss of grey matter integrity and volume in the temporal limbic system proportionate to symptomatology.

Phase and total duration of illness have also been shown to be associated with subcortical volumetrics in MDD, with limbic brain regions generally considered to be more structurally affected in persistent forms of MDD^64^. While early depression is associated with increased amygdala volume, with greater disease duration the amygdala volume declines^65,66^. The number of neurovascular cells in the accessory basal nucleus of the amygdala also appears to be associated with duration of depression, so that MDD patients with a disease duration of under 5 years exhibit significantly more cells than individuals with an MDD duration of 5 years or more^67^. Age plays an additional important role in hippocampal and amygdala volumetrics, neurochemistry and functionality, as well as serving as a source of variability in both in MDD and healthy controls^68,69^. Our results however do not suggest a relationship between any amygdala nuclei or hippocampal subfield volume and duration of depressive episode. This may be due to a lack of sufficient power in the present pilot sample to detect smaller effect sizes. Alternatively, variability within the depressed sample may have masked possible volumetric associations with duration.

Another likely possibility is that vulnerability to mood disorders may be conferred developmentally as a latent trait in hippocampal and amygdala volumetrics^70^. Animal models showing higher risk endophenotypes for MDD have been linked to hippocampal and amygdala remodeling^62^. One of the largest studies of amygdala volumes in MDD demonstrated increased volumes in depressed females without family history of MDD compared to depressed females with family history^20^ and population-based study utilizing polygenic risk has also suggested that hippocampal volume and MDD share a genetic basis^7^. The possibility of a genetic predisposition to reduced hippocampal and amygdala subvolumes in individuals susceptible to MDD likely mediates the known outcomes of HPA axis dysregulation and BDNF reduction, precipitating an association between decreasing subfield and nuclei size and increasing severity of depressive symptoms.

Despite the statistically stringent methodology used in the present study, some limitations of our results should be noted. Firstly, a small pilot sample was used, restricted in part by increased safety criteria at 7T field-strength. A meta-analysis of subcortical volume alterations in depression reports high sample size necessity for detection of between group differences in hippocampal volume, therefore lack of significant differences in the present study should be interpreted with caution^17^. Additionally, amygdala nuclei and hippocampal subfields exhibit collinearity which is inherent to brain anatomy (Supplementary Fig. 2), and this may have been a confounding factor in our statistical analysis of the dataset. Nevertheless, due to the survival of many of our significant correlative results after FDR correction, in addition to adjusting for interacting factors, our results remain promising. The clinical evaluation of depressive symptoms utilized a robust scale, the MADRS, which provides a measure of MDD severity^71^. However, the majority of participants did not fill out the MADRS questionnaire on the day of the scan, which may be considered to be a weakness when examining its associations with structural brain measures. Another limitation is that the duration of illness within our sample was varied, and existing research has suggested the existence of structural differences in the brain in long-term and short-term MDD^39^. However, when examined as a regressor the current sample showed no association between illness duration and any hippocampal or amygdala region volume, possibly due to the statistical control of age^18^. Also, though no participants were taking antidepressant medication at the time of scanning and clinical evaluation, it should be noted that a number of participants had taken antidepressant medication in the past, including individuals with a number of trials suggestive of possible treatment-resistant depression, which may or may not of influenced the present findings. Additionally, *in vivo* structural MRI alone cannot accurately predict biological or neuronal level causes of the identified sensitivity of the amygdala nuclei and hippocampal subfields volumes to depressive symptomatology, therefore potential mechanisms behind our findings should be treated cautiously. To the authors knowledge, the FreeSurfer hippocampal-amygdala segmentation technique has also not been independently validated. Whilst we acknowledge that FreeSurfer segmentations can fluctuate in their robustness and accuracy across field strengths^72^, the technique applied to the current data was developed at 7T using postmortem tissue and performance was analyzed at 3T on large datasets^43^. The analysis was therefore deemed suitable for the present 7T images, however further validation of this methodology will be a helpful future addition to the field.

We believe that this study’s strengths eclipse the relative limitations. Specifically, the novel use of ultra high-field 7T MRI to investigate the limbic region of MDD patients offers superior signal and resolution over conventional field strength imaging, enabling improved high resolution at the level of nuclei and subfields. Moreover, the utilization of automated segmentation methods is beneficial for possible future replication and offers some control over investigator bias. In all, we present novel findings that the amygdala nuclei and hippocampal subfields exhibit an almost uniform volumetric reactivity to the severity of depressive symptoms.

## Supplementary information

Supplementary Information

## Data Availability

The datasets generated and analyzed for the current study can be made available from the corresponding author upon reasonable request.
