# Supplementary material for: Structural MRI at 7T reveals amygdala nuclei and hippocampal subfield volumetric association with Major Depressive Disorder symptom severity
Source: Sci Rep. 2019 Jul 15;9:10166. doi: 10.1038/s41598-019-46687-7 (PMC6629636; doi:10.1038/s41598-019-46687-7)
Supplement: Supplementary file 1 — Supplementary Information [file 41598_2019_46687_MOESM1_ESM.docx]

**Structural MRI at 7T reveals amygdala nuclei and hippocampal subfield volumetric association with Major Depressive Disorder symptom severity**

S. S. G. Brown, J. W. Rutland, G. Verma, R. E. Feldman, J. Alper, M. Schneider, B. N. Delman, J. M. Murrough, P. Balchandani

**
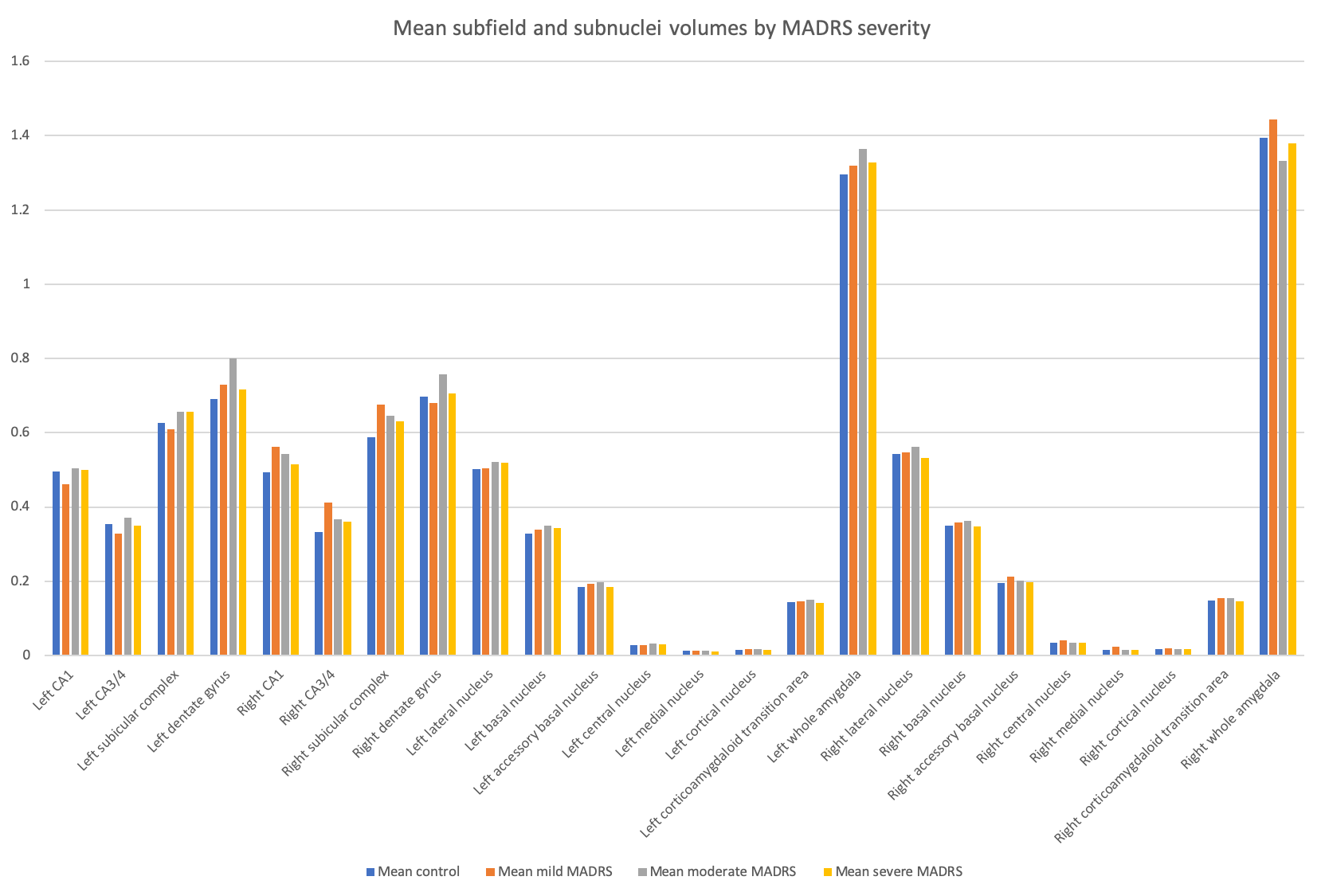
**

**Supplementary Fig. S1.** Amygdala subnuclei and hippocampal subfield mean volumes as grouped by MADRS severity

**Supplementary Fig. S2.** Correlation coefficients of volumetric measurements, ordered by first principle component, indicating degree of collinearity between hippocampal subfields and amygdala nuclei.
